# SAR Image Segmentation Using Voronoi Tessellation and Bayesian Inference Applied to Dark Spot Feature Extraction

**DOI:** 10.3390/s131114484

**Published:** 2013-10-25

**Authors:** Quanhua Zhao, Yu Li, Zhenggang Liu

**Affiliations:** 1 Institute for Remote Sensing Science and Application, School of Geomatics, Liaoning Technical University, Fuxin 123000, China; E-Mails: zhaoquanhua@lntu.edu.cn (Q.Z.); liuzhenggang@lntu.edu.cn (Z.L.); 2 The Key Laboratory of Marine Oil Spill Identification and Damage Assessment Technology, North China Sea Environmental Monitoring Center, State Oceanic Administration, Qingdao 266033, China

**Keywords:** Voronoi tessellation, Bayesian inference, feature extraction, oil spill, dark spots

## Abstract

This paper presents a new segmentation-based algorithm for oil spill feature extraction from Synthetic Aperture Radar (SAR) intensity images. The proposed algorithm combines a Voronoi tessellation, Bayesian inference and Markov Chain Monte Carlo (MCMC) scheme. The shape and distribution features of dark spots can be obtained by segmenting a scene covering an oil spill and/or look-alikes into two homogenous regions: dark spots and their marine surroundings. The proposed algorithm is applied simultaneously to several real SAR intensity images and simulated SAR intensity images which are used for accurate evaluation. The results show that the proposed algorithm can extract the shape and distribution parameters of dark spot areas, which are useful for recognizing oil spills in a further classification stage.

## 1. Introduction

Oil spills from operational discharges and ship accidents always have calamitous impacts on the marine environment and ecosystems, even with small oil coverage volumes. Remote sensing solutions using space-borne or airborne sensors are playing an increasingly important role in monitoring, tracking and measuring oil spills and are receiving much more attention from governments and organizations around the world. Compared to airborne sensors, satellite sensors, with their large extent observation, timely data available and all weather operation, are proven to be more suitable for monitoring oil spills in marine environments, whilst the latter can be easily used to identify polluters and oil spill types but are of limited use due to costs and weather conditions [1]. Currently, the commonly used SAR sensors for this purpose include RADARSAT-1/2, ENVISAT, ERS-1/2, and so on [1–4]. The detectability of oil spills by SAR sensors is based on the fact that oil slicks dampen the Bragg waves on the ocean surface and reduce the radar backscatter coefficient. Unfortunately, other physical phenomena, for example, low-wind areas, wind-shadow areas near coasts, rain cells, currents, upswelling zones, biogenic films, internal waves, and oceanic or atmospheric fronts, can also generate dark areas, known as look-alikes, in SAR images [5,6]. Another factor which influences the backscatter level and the visibility of oil slicks on the sea surface is the wind level. Oil slicks are visible only for a limited range of wind speeds [4,6].

Generally speaking, oil spill recognition includes three stages: dark spot detection, dark spot feature extraction and oil spill classification [6–8]. The work in this paper focuses on the feature extraction of detected dark spots [9]. The task at this stage involves defining and acquiring the features existing in SAR images, which can be efficiently used in the classification stage to distinguish oil spills from look-alikes. Commonly defined features for this purpose include the geometry and shape of the dark spot area, textures, contrast between dark spots and their surroundings, and dark spot contextual information [6,7,10,11]. In this paper, a new segmentation-based approach to extract the areas and outlines of dark regions and Gamma distribution parameters of dark regions in SAR images is presented. Many researchers have focused on their work on SAR segmentation issues. The segmentation algorithms for dark spot detection include threshold segmentation [12,13], edge extraction based segmentation, wavelets [14], and fuzzy clustering [15]. SAR images are highly speckled due to coherent processing [16]. The analysis of SAR images is usually required for region and statistics-based methodology in order to reduce the speckle effect. Following this idea, a new algorithm for segmentation of SAR images is considered, which is based on Voronoi tessellation [17] and Bayesian inference [18–21]. Voronoi tessellation has been widely used to characterize models for many natural phenomena or processes in crystallography, metallography, physics, astrophysics, biology, ecology, geology, geography, *etc.* [17] To segment a SAR intensity image, it is reasonable to approximate the homogenous regions in an SAR image by Voronoi polygons. The number of Voronoi polygons is assumed unknown. The intensities of pixels within a region defined by the polygons are distributed according to identical and independent Gamma distribution, with the parameters dependent on the homogenous region to which the polygon belongs. Following the Bayesian paradigm, the mathematical form of a posterior distribution is obtained up to an integrating constant. In order to simulate the posterior, a Markov Chain Monte Carlo (MCMC) scheme is developed, in which the move types are designed, including: (1) updating the distribution parameters; (2) updating the label for a polygon which indicates a homogenous region including the polygon; (3) updating the generating point and birth and death of generating points. For simplicity, a Maximum A Posteriori (MAP) criterion is used to obtain the optimal image segmentation and feature extraction.

The paper is organized as follows: Section 2 describes in detail the algorithm developed in this paper. In Section 3, the proposed algorithm is tested by several simulated 4-looks SAR intensity images and applied to RADARSAT-1 SAR intensity images for oil spill extraction. Finally, Section 4 contains concluding remarks and perspectives for further research.

## 2. Description of the Proposed Algorithm

### 2.1. Image Model

In a spatial statistic model, SAR backscatter energy can be characterized by a random field defined on a domain ***D***⊂*R*^2^, {*Z*(*x, y*); (*x, y*) ∈ ***D***}, where *Z*(*x, y*) is a random function defined at the location (*x, y*). Under this framework, a SAR intensity image with *n* pixels can be viewed as the set of spatial samples from the random field at *n* discrete sites. A conventional sampling scheme is uniform sampling, in which regularly spaced positions are used. Therefore, a conventional digital SAR intensity image can be expressed by a set of random variables, that is, ***Z*** = {*Z_i_* = *Z*(*x_i_, y_i_*); (*x_i_, y_i_*) ∈ ***D***, *i* = 1, …, *n*} where *i* is the index of pixels, (*x_i_, y_i_*) is the location of pixel *i, Z_i_* is the intensity variable of pixel *i*. The basic idea behind the segmentation algorithm for dark spot feature extraction lies in partitioning ***D*** into two homogenous regions ***D***_1_ and ***D***_2_ corresponding to the dark spot areas and its surroundings, respectively. To this end, ***D*** is partitioned into *m* sub-regions, that is, ***D*** = {*P_j_*; *j* = 1,.., *m*}, where *m* is assumed unknown and with a prior distribution *p*(*m*), and a label *L_j_* ∈ {1, 2} is assigned to the sub-region, say *P_j_*, to indicate the homogenous region to which *P_j_* belongs. The set of labels for all sub-regions forms a label field, that is, ***L*** = {*L_j_*; *j* =1, …, *m*}. The intensities of pixels included in a sub-region are assumed to satisfy identical and independent Gamma distribution with parameters in terms of its label, so the joint probability distribution for the intensities of all pixels in the sub-region *P_j_*, ***Z****_j_* = {*Z_i_*; (*x_i_, y_i_*) ∈ *P_j_*}, can be written as:

(1) $$p(Z_{j})=\underset{(x_{i},y_{i})\in P_{j}}{\text{∏}}\frac{{Z_{i}}^{\alpha_{{L_{j}}^{-1}}}}{\Gamma (\alpha_{L_{j}}){\beta_{L_{j}}}^{\alpha_{L_{j}}}}\cdot \exp\left(-\frac{Z_{i}}{\beta_{L_{j}}}\right)$$

where (*x_i_, y_i_*) ∈ *P_j_*, Γ(·) is Gamma function, *α* and *β* are the shape and scale parameters of the Gamma distribution. Let ***Θ*** = {*α*_1_, *α*_2_, *β*_1_, *β*_2_} be the set of parameters for Gamma distributions corresponding to dark spill regions and its surroundings, respectively. For a flexible and convenient tessellation, the Voronoi tessellation [17,22] is used for partitioning ***D*** into sub-regions called Voronoi polygons. Those polygons are specified by *m* generating points located at (*u_j_, v_j_*) ∈ ***D***, where (*u_j_, v_j_*) are assumed to be independently distributed on ***D*** with a probability density function *p*(*u_j_, v_j_*). Let ***G*** = {(*u_j_, v_j_*) ∈ ***D***; *j* = 1, …, *m*} denotes the set of all generating points. Given ***G***, the Voronoi tessellation partitions ***D*** into a set of polygons, that is, ***D*** = {*P_j_*; *j* = 1, …, *m*}, in which the *j*th Voronoi polygon *P_j_* associated with the generating point (*u_j_, v_j_*) is comprised of the points nearest to (*u_j_, v_j_*) than to others in ***G***, that is:

(2) $$P_{j}=\left\{\left(x,y\right)\in D;\left|\left(x,y\right)-\left(u_{j},v_{j}\right)\right|<\underset{\left(u_{j^{\prime }},v_{j^{\prime }})\in G/(u_{j},v_{j}\right)}{\min}\left|\left(x,y)-(u_{j'},v_{j'}\right)\right|\right\}$$

Given ***Θ***, ***L***, ***G*** and *m*, ***Z*** can be characterized by the likelihood function, *p*(***Z*** |***Θ***, ***L***, ***G***, *m*), as follows:

(3) $$p(Z|\Theta ,L,G,m)=\overset{m}{\underset{j=1}{\text{∏}}}\underset{(x_{i},y_{i})\in P_{j}}{\text{∏}}\frac{Z_{i}^{\alpha_{L_{j}}-1}}{\Gamma (\alpha_{L_{j}}){\beta_{L_{j}}}^{\alpha_{L_{j}}}}\cdot \exp\left(-\frac{Z_{i}}{\beta_{L_{j}}}\right)$$

### 2.2. Bayesian Model

Using a Bayesian paradigm, the inference about parameters {***Θ***, ***L***, ***G***, *m*} given ***Z*** will be determined based on the joint posterior *p* (***Z*** |***Θ***, ***L***, ***G***, *m*), which can be written as:

(4) p(Θ,L,G,m∣Z)∝p(Z∣Θ,L,G,m)⋅p(Θ)⋅p(L∣m)⋅p(G∣m)p(m)

where *p*(***Z*** |***Θ***, ***L***, ***G***, *m*) is the likelihood defined by Equation (3)*p*(***Θ***) is the prior distribution for Gamma distribution parameters. Under the assumptions that all distribution parameters are independent, have *p*(***Θ***) = *p*(*α*_1_)*p*(*α*_2_)*p*(*β*_1_)*p*(*β*_2_). Furthermore, assume that the scale (resp. shape) parameters are drawn from Gaussian distribution with mean *μ_β_* (resp. *μ_α_*) and standard deviation *σ_β_* (resp. *σ_α_*), that is, *β ∼ N*(*μ_β_σ_β_*) (resp. *α* ∼ *N*(*μ_α_*,*σ_α_*)). As a result, the probability distribution function *p*(***Θ***) can be expressed as:

(5) $$p(\Theta )=\overset{2}{\underset{k=1}{\text{∏}}}\frac{1}{\sqrt{2\pi }\sigma_{\alpha }}\exp\left(-\frac{{\left(\alpha_{k}-\mu_{\alpha }\right)}^{2}}{2{\sigma_{\alpha }}^{2}}\right)\times \overset{2}{\underset{k=1}{\text{∏}}}\frac{1}{\sqrt{2\pi }\sigma_{\beta }}\exp\left(-\frac{{\left(\beta_{k}-\mu_{\beta }\right)}^{2}}{2{\sigma_{\beta }}^{2}}\right)$$

*p*(***L***|*m*) is the prior distribution for label field which characterizes the relationship among labels. In this paper, the label field is modeled by a Markov Random Field [23], and an improved stationary Potts model [24] with 2 labels is used to model the prior distribution *p*(***L***|*m*). Two Voronoi polygons *P_j_* and *P_j'_* are neighbors if and only if they have a mutual boundary, denoted *P_j_* ∼ *P_j_*_'_. For a polygon *P_j_* with label *L_j_*, given its neighboring polygons *NP_j_* = { *P_j_*_'_; *P_j_*_'_ ∼ *P_j_* }, the conditional distribution of *L_j_* on {*L_j_*_'_; *P_j_*_'_ ∈ *NP_j_* } is expressed as:

(6) $$p(L_{j}|L_{j'}\in NP_{j})=\frac{\exp\left\{b\underset{P_{j'}\in NP_{j}}{\text{∑}}t(L_{j},L_{j'})\right\}}{\overset{2}{\underset{L=1}{\text{∑}}}\exp\left\{b\underset{P_{j\prime }\in NP_{j}}{\text{∑}}t(L_{j},L_{j'})\right\}}$$

where *b* > 0 is the constant to control the neighborhood dependences, and *t* (*x, y*) = 1, if *x* = *y*, otherwise *t* (*x, y*) = 0. As a result, the prior distribution *p*(***L****| m*) can be written as:

(7) $$p(L|m)=\overset{m}{\underset{j=1}{\text{∏}}}p(L_{j^{\prime }}|L_{j^{\prime }},P_{j^{\prime }}\in NP_{j})$$

Assume that the locations of generating points (*u_j_, v_j_*) are independent and drawn from ***D*** uniformly, so the prior distribution *p* (***G*** | *m*) is:

(8) $$p(G|m)=\overset{m}{\underset{j=1}{\text{∏}}}\left(\frac{1}{\left|D\right|}\right)={\left(\frac{1}{\left|D\right|}\right)}^{m}$$

where |***D***| is the area of ***D***. In this paper, the number of generating points *m* is assumed to satisfy a Poisson distribution with mean *λ*, that is:

(9) $$p(m)=\frac{\lambda^{m}}{m!}\exp(-\lambda )$$

The posterior distribution defined in Equation (4) can be derived according to the prior distributions of Equations (5)–(9) and image model in Equation (3) as follows:

(10) $$\begin{array}{l} p(\Theta ,L,G,m|Z)\propto \overset{m}{\underset{j=1}{\text{∏}}}\underset{(x_{i},y_{i})\in D_{j}}{\text{∏}}\frac{{Z_{i}}^{\alpha_{{L_{j}}^{-1}}}}{\Gamma (\alpha_{L_{j}}){\beta_{L_{j}}}^{\alpha_{L_{j}}}}\exp\left(-\frac{z_{i}}{\beta_{L_{j}}}\right)\times \overset{2}{\underset{k=1}{\text{∏}}}\frac{1}{\sqrt{2\pi }\sigma_{\alpha }}\exp\left(-\frac{{\left(\alpha_{k}-\mu_{\alpha }\right)}^{2}}{2{\sigma_{\alpha }}^{2}}\right)\times \\ \overset{2}{\underset{k=1}{\text{∏}}}\frac{1}{\sqrt{2\pi }\sigma_{\beta }}\exp\left(-\frac{{\left(\beta_{k}-\mu_{\beta }\right)}^{2}}{2{\sigma_{\beta }}^{2}}\right)\times \overset{m}{\underset{j=1}{\text{∏}}}\frac{\exp\left\{b\underset{P_{j'}\in NP_{j}}{\text{∑}}t(L_{j},L_{j^{\prime }})\right\}}{\overset{2}{\underset{l=1}{\text{∑}}}\exp\left\{b\underset{P_{j'\in NP_{j}}}{\text{∑}}t(L,L_{j'})\right\}}\times {\left(\frac{1}{\left|D\right|}\right)}^{m}\times \frac{\lambda^{m}}{m!}\exp(-\lambda ) \end{array}$$

### 2.3. Simulation

In order to segment an SAR intensity image, it is necessary to simulate it from the posterior distribution in Equation (10) and to estimate its parameters. Let ***Φ*** = (***Θ***, ***L***, ***G***, *m*) be parameter vectors. It is worthy to note that when *m* is variable, the dimension of the parameter vector ***Φ*** is varied. In this paper, Reversible Jump Markov Chain Monte Carlo (RJMCMC) algorithm [21] is used for this simulation. According to [21], at each iteration a new candidate Φ^*^ for ***Φ*** is proposed by an invertible deterministic function Φ^*^ = Φ^*^(***Φ***, ***s***) (assume that the dimension of Φ^*^ is higher than that of ***Φ***). ***s*** is a random vector defined for accomplishing a transition from (***Φ***, ***s***) to Φ^*^ with dimension matching, that is, |***Φ***| + |***s***| = |Φ^*^|. The appropriate acceptance probability for the proposed transition is given by:

(11) $$\alpha (\Phi ,\Phi^{*})=\min\left\{1,\frac{p(\Phi^{*}|Z)r(\Phi^{*})}{p(\Phi |Z)r(\Phi )q(\text{s})}\left|\frac{\partial (\Phi^{*})}{\partial (\Phi ,s)}\right|\right\}$$

where *q*(***s***) is the density function of ***s*** and *r*(Φ^*^) and *r*(***Φ***) are the probabilities of a given move type in the states Φ^*^ and ***Φ***, respectively. The Jacobian |∂(Φ^*^)/∂(***Φ***, ***s***)| arises from the change of variable from (***Φ***, ***s***) to Φ^*^. The move types designed in this paper include: (1) updating Gamma distribution parameters; (2) updating the labels; (3) updating the positions of generating points; (4) birth or death of generating points.

*Move* 1: *updating the gamma distribution parameters*. Rewriting ***Θ*** = {*Θ_k_, k* = 1, 2} where *Θ_k_* = (*α_k_, β_k_*). Assume that the probability distributions for the proposals α_k_^*^ and β_k_^*^ are Gaussian distributions with means *α_k_* and *β_k_*, and standard deviations *ε_αk_* and *ε_βk_*, respectively, that is, α_k_^*^ ∼ *N* (*α_k_., ε_αk_*) and β_k_^*^ ∼ *N* (*β_k_, ε_βk_*). The acceptance probability for the proposals α_k_^*^ and β_k_^*^ can be obtained by:

(12) $$a_{1}(\Theta k,{\Theta_{k}}^{*})=\min\left\{1,\underset{j\in J_{k}}{\text{∏}}\frac{\underset{(x_{i},y_{i})\in P_{j}}{\text{∏}}p(Z_{i}|{\Theta_{k}}^{*})\times p({\Theta_{k}}^{*})}{\underset{(x_{i},y_{i})\in P_{j}}{\text{∏}}p(Z_{i}|\Theta_{k})\times p(\Theta_{k})}\right\}$$

where *J_k_* = {*j*', *L_j_*_'_ = *k*}, *k*∈ {1, 2}.

*Move* 2: *updating labels*. A label randomly drawn from the label fields ***L***, say *L_j_*, is updated by proposing a new label L_j_^*^, which is uniformly drawn from {1, 2}. The acceptance probability for L_j_^*^ can be written as:

(13) $$a_{2}(L_{j},{L_{j}}^{*})=\min\left\{1,\frac{\underset{(x_{i},y_{i})\in P_{j}}{\text{∏}}\frac{{Z_{i}}^{\alpha_{{L_{j}}^{*}}-1}}{\Gamma (\alpha_{{L_{j}}^{*}}){\beta_{{L_{j}}^{*}}}^{\alpha_{{L_{j}}^{*}}}}\exp\left(-\frac{z_{i}}{\beta_{{L_{j}}^{*}}}\right)\times \exp\left(2b\underset{P_{j'}\in NP_{j}}{\text{∑}}t({L_{j}}^{*},L_{j'})\right)}{\underset{(x_{i},y_{i})\in P_{j}}{\text{∏}}\frac{{Z_{i}}^{\alpha_{L_{j}}-1}}{\Gamma (\alpha_{L_{j}}){\beta_{L_{j}}}^{\alpha_{L_{j}}}}\exp\left(-\frac{z_{i}}{\beta_{L_{j}}}\right)\times \exp\left(2b\underset{P_{j\prime }\in NP_{j}}{\text{∑}}t(L_{j},L_{j'})\right)}\right\}$$

*Move* 3: *moving the position of generating points*. One of generating points in ***G*** is drawn at random, say (*u_j_, v_j_*). A proposed position is (u_j_^*^, v_j_^*^) by drawing uniformly in *P_j_*. The new proposed position gives rise to the local changes of *P_j_* and its neighbour polygons to {P_j'_^*^, NP_j_^*^}. The acceptance probability for the move turns out to be:

(14) $$a_{3}\left({\left(u_{j},v_{j}\right)}_{j},({u_{j}}^{*},{v_{j}}^{*})\right)=\min\left\{1,\frac{\underset{P_{j'}\in \{P_{j},NP_{j}^{*}\}}{\text{∏}}\underset{(x_{i},y_{i})\in P_{j'}*}{\text{∏}}\frac{Z_{i}^{\alpha_{L_{j}}-1}}{\Gamma (\alpha_{L_{j}}){\beta_{L_{j}}}^{\alpha_{L_{j}}}}\exp\left(-\frac{z_{i}}{\beta_{L_{j}}}\right)}{\underset{P_{j'}\in \{P_{j},NP_{j}\}}{\text{∏}}\underset{(x_{i},y_{i})\in P_{j'}}{\text{∏}}\frac{Z_{i}^{\alpha_{L_{j}}-1}}{\Gamma (\alpha_{L_{j}}){\beta_{L_{j}}}^{\alpha_{L_{j}}}}\exp\left(-\frac{z_{i}}{\beta_{L_{j}}}\right)}\right\}$$

**Figure 1.**
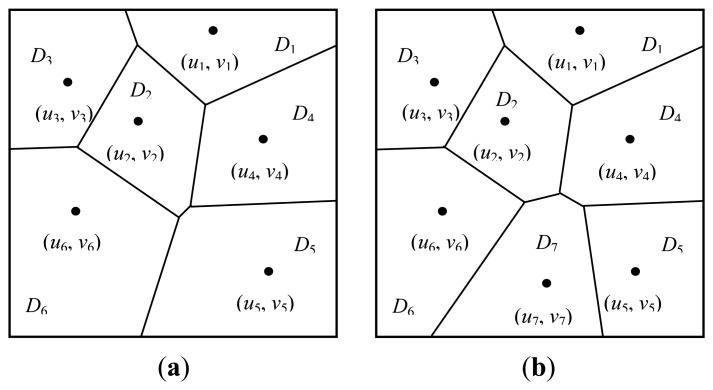
(**a**) Voronoi tessellation with six polygons *D*_1_–*D*_6_ corresponding to generating points (*u*_1_, *v*_1_)–(*u*_6_, *v*_6_); (**b**) Voronoi tessellation with seven polygons *D*_1_–*D*_7_ formed by adding the generating point (*u*_7_, *v*_7_).

*Move* 4: *birth or death of generating points*. Suppose that the current number of generating points is *m* and let the probabilities of proposing a birth or death operation be *b_m_* or *d_m_*, respectively. Consider a birth operation which increases the number of generating points from *m* to *m*+1 and assume that the new generating point is labelled with *m* +1 and its location (*u_m_*_+1_, *v_m_*_+1_) is drawn uniformly from *D*. Let the polygon induced by (*u_m_*_+1_, *v_m_*_+1_) be *P_m_*_+1_ and the set of labels of *P_m_*_+1_'s neighbor polygons is *N_m_*_+1_. The Voronoi tessellation is modified by the addition of this generating point from *P* = {*P*_1_, …, *P_j'_*, …, *P_m_*} to *P* = {*P*_1_, …, P_j'_^*^, …, *P_m_, P_m_*_+1_}. Figure 1 shows the modified Voronoi tessellation by the addition of a new generating point, in which the original tessellation have six generating points and they induce six polygons, see Figure 1a. By proposing a new generating point labelled 7, a new polygon is generated by it and denoted *P*_7_. It can be observed from Figure 1b that the neighbors of *P*_7_ include *P*_2_, *P*_4_, *P*_5_ and *P*_6_, that is, *NP*_7_ = {2, 4, 5, 6}.

The new label *L_m_*_+1_ for polygon *P_m_*_+1_ is uniformly drawn from {1, 2}. It is evident that a birth or a death of generating point does not affect the Gamma distribution parameters in ***Θ***. As a result, the parameter vector for the birth operation becomes Φ^*^ = (***Θ***, L^*^, G^*^, *m*+1) where L^*^ = (*L*_1_, …, *L_m_, L_m_*_+1_), G^*^ = (***G***, (*u_m_*_+1_, *v_m_*_+1_)). The acceptance probability of the birth can be written as:

(15) $$a_{4b}(\Phi ,\Phi^{*})=\min\{1,R_{4b}\}$$

where:

(16) $$\begin{array}{c} R_{4b}=\frac{p(Z|\Theta ,L^{*},G^{*},m+1)p(m+1)p(L^{*}|m+1)p(G|m+1)r_{d_{m+1}}(\Phi^{*})}{p(Z|\Theta ,L,G,m)p(m)p(L|m)p(G|m)r_{b_{m}}(\Phi )q(s)}\left|\frac{\partial (\Phi^{*})}{\partial (\Phi ,s)}\right|\\ =\frac{\underset{P_{j}\in \{P_{m+1},NP_{m+1}\}}{\text{∏}}\underset{(x_{i},y_{i})\in P_{j}}{\text{∏}}\frac{Z_{i}^{\alpha_{L_{j}}-1}}{\Gamma (\alpha_{L_{j}}){\beta_{L_{j}}}^{\alpha_{L_{j}}}}\exp(-\frac{z_{i}}{\beta_{L_{j}}})}{\underset{P_{j}\in NP_{m+1}}{\text{∏}}\underset{(x_{i},y_{i})\in P_{j}}{\text{∏}}\frac{Z_{i}^{\alpha_{L_{j}}-1}}{\Gamma (\alpha_{L_{j}}){\beta_{L_{j}}}^{\alpha_{L_{j}}}}\exp\left(-\frac{z_{i}}{\beta_{L_{j}}}\right)}\times \frac{\underset{P_{j\in \{Pm+1,NP_{m+1}\}}}{\text{∏}}\frac{\exp\left(b\underset{P_{j'\in N{P_{j}}^{*}}}{\text{∑}}t(L_{j},L_{j'})\right)}{\overset{2}{\underset{k=1}{\text{∑}}}\exp\left(b\underset{P_{j'\in N{P_{j}}^{*}}}{\text{∑}}t(k,L_{j'})\right)}}{\underset{P_{j}\in NP_{m+1}}{\text{∏}}\frac{\exp\left(b\underset{P_{j'\in NP_{j}}}{\text{∑}}t(L_{j},L_{j'})\right)}{\overset{2}{\underset{l=1}{\text{∑}}}\exp\left(c\underset{P_{j'\in NP_{j}}}{\text{∑}}t(l,l_{j'})\right)}}\times \frac{\lambda }{{\left(m+1\right)}^{2}*k*|} \end{array}$$

where *r_bm_* = *b_m_, r_dm_*_+1_ = *d_m_*_+1_/(*m*+1), ***s*** = *l_m_*_+1_. The acceptance probability of a death of generating point is:

(17) $$\alpha_{4d}(\Phi ,\Phi^{*})=\min\{1,R_{4d}\},\;\text{and}\;R_{4d}={R_{4b}}^{-1}$$

For any given proposal with acceptance probability *a*, it is accepted if and only if *a* ≥ *ψ*, where *ψ* is drawn from [0, 1] uniformly, that is, *ψ* ∼ *U* (0, 1).

### 2.4. Optimization

To estimate the parameter vector ***Θ***, its samples are drawn from the above posterior distribution in Equation (10) using the RJMCMC scheme. An MAP criterion is used to obtain the final segmentation, which is represented by the optimal label filed under maximizing the posterior. The MAP estimator is designed as:

(18) $$\hat{L}=\arg\{\max(p(\Theta ,L,G,m|Z))\}$$

## 3. Experimental Results and Discussion

To assess the accuracy of the proposed algorithm for extracting features of dark spots, two types of data, 4-looks SAR intensity images and simulated SAR intensity images which simulate 4-looks SAR intensity images, are used. It has been shown that multilook SAR intensity images can be modeled by Gamma distributions with fixed scale parameters, which are equal to the number of looks.

Table 1 gives the constants used in this experiment, where *λ* is the mean of Poisson distribution from which the number of generating points is drawn. In a certain range, the value of *λ* does not affect the segmentation results. For simplicity, the correlation coefficient *b* is set as 1. The constants *μ_α_* and *μ_β_* are the means of shape parameter *α* and scale parameter *β* of the Gamma distributions in Equation (5), respectively, *i.e., μ_α_*= E(*α*) and *μ_β_* = E(*β*). Given a multi-look SAR image in which the intensities of pixels are characterized by Gamma distribution, *α* is equal to the number of its looks. In this paper, since *α* is considered as a random variable the value *μ_α_* is set as the number of looks. For Gamma distribution with parameters *α* and *β*, the product of the two parameters, *αβ*, is equal to its mean. Then the value *μ_α_× μ_α_* = E(*α*)E(*β*) = E(*αβ*) (the last equation is true, since *α* and *β* are independent) is taken 128 = 256/2 (*i.e.*, the midpoint of 256 grey levels) since the pixel intensities in a grey-scale image vary in the range of 0 and 255. *ε_α_* and *ε_β_* are the standard deviations for the proposal densities of the shape and scale parameters, that is, α^*^ ∼ *N*(*α^t^, ε_α_*) and β^*^ ∼ *N*(*β^t^, ε_β_*) where α^*^ and β^*^ is the proposals, *α^t^* and *β^t^* are the shape and scale parameters at *t* th iteration. They affect the sampling and the convergence of the algorithm under the MCMC scheme [18] suggested choosing the proposal variances so that the acceptance probability lies in the interval (0.3–0.7). However we have found that the proposal variances causing the acceptance probability around 0.1 still make the algorithm work well. *T* is the number of iterations. Usually, it depends on the complexity of the scene revealed in a SAR image and requirement of segmentation accuracy.

### 3.1. Simulated SAR Imagery

**Figure 2.**
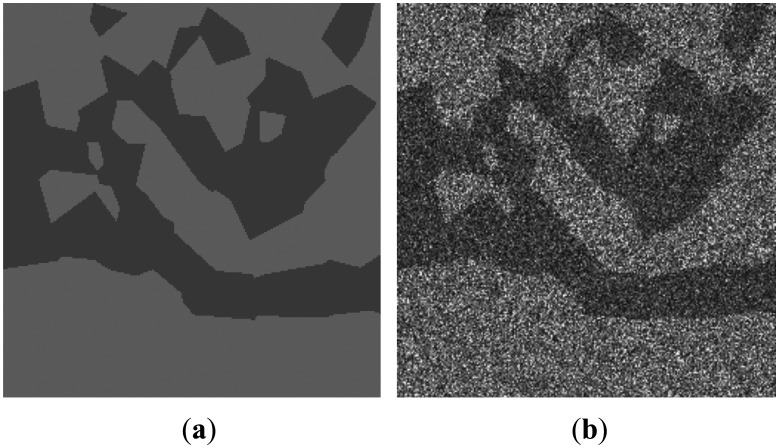
Results of (**a**) the partition of domain; (**b**) the simulated SAR image.

Figure 2 shows a simulated SAR image, which is generated based on the partition of a domain as shown in Figure 2a. In the simulated image in Figure 2b, the intensity values of pixels in each homogeneous region are drawn from gamma distributions with shape parameters equal to 4, and the scale parameters equal 18 and 28 for dark spill and background classes, respectively.

Table 2 gives the experimental results after 4,000 iterations of the proposed algorithm, including estimated distribution parameters α and *β*, and the estimation errors for the parameters (*e*%) corresponding to the optimal segmentations.

**Figure 3.**
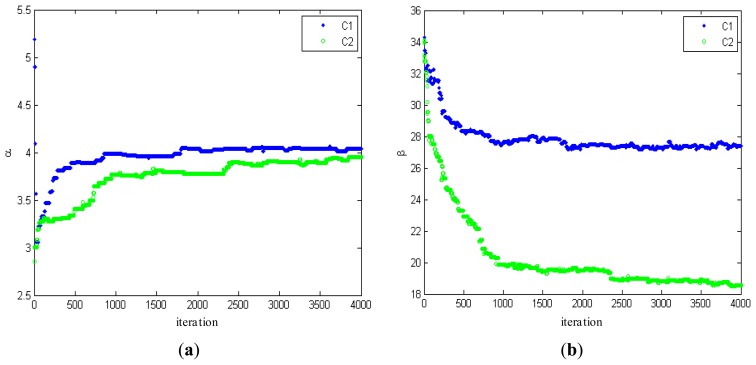
Changes of (**a**) estimated shape parameters and (**b**) scale parameters during 4,000 iterations.

Figure 3 shows the changes of estimated parameters. From Figure 3, it can be seen that the estimated parameters converge to their stable values well at around 1,000 iterations, consistent with the results from the testing on a number of SAR intensity mages. In order to illustrate the convergence and stability of the proposed algorithm, 4,000 iterations are carried out.

**Figure 4.**
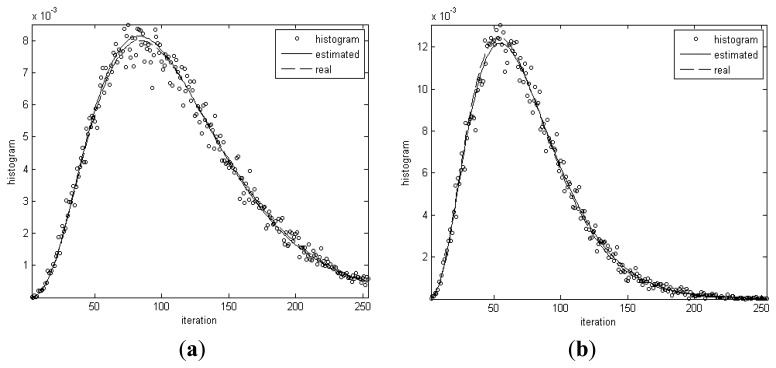
Histogram and curves of gamma distributions with real and estimated model parameters.

Figure 4 shows the histograms for the homogenous regions in the simulated image and distribution curves of Gamma distributions with real and estimated parameters, respectively. From Table 2 and Figure 4, it can be seen that the real and estimated values of distribution parameters for dark spot regions are very close. The maximum error is only 2.8%. The conclusion can be drawn that the algorithm has the capability of extracting the distribution parameters from data.

**Figure 5.**
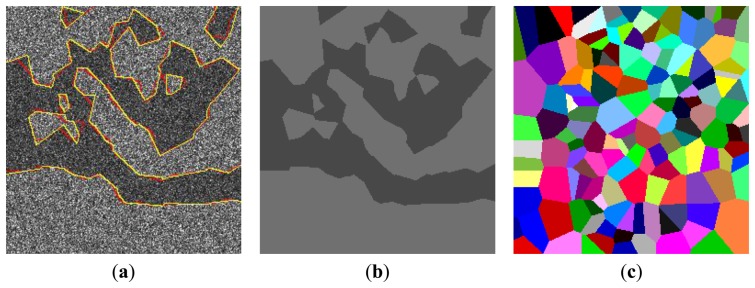
Results of (**a**) final partition of image domain; (**b**) optimal segmentation after 4,000 iterations and Outlines of the real regions (yellow) and segmented regions (red) overlaid on the simulated image.

Figure 5 shows the final partitions with 152 polygons. Figure 5a, b gives the optimal segmentations obtained at 4,000 iterations, in which the segmented homogeneous regions are represented by their mean. To visually assess the accuracy for extracted dark spot regions, the extracted outlines of the segmented image in red and real outlines which is used for simulating image in yellow are both overlaid on the original images, see Figure 5c. Visually, the outlines of extracted oil spill regions(yellow) match their real outlines(red) very well.

In this experiment, two evaluation schemes are used to assess the accuracy of extracted oil spill area quantitatively. First of all, some of the common measures are used for accuracy assessment, including error matrix, producer's accuracy, consumer's accuracy, overall and Kappa coefficient. Table 3 gives the error matrixes which compare the segmented homogeneous regions as thematic classes to the real homogenous regions as reference.

The entries in the matrix contain a count of pixels, which is based on the labels assigned to pixels in both the segmented image and the synthesizing images. For example, if a pixel is segmented to the oil spill region with the label 1 and actually belongs to the water region with label 2, it will be counted in the error matrix entry of column 1 and row 2, denoted by *C*_12_. The values of diagonal entries represent the count of correctly segmented pixels. The Table also lists row total (Σ*C._r_*) and column total (Σ*C_s_*.) which account for the total count of pixels segmented to regions *r* and belonging to regions *s*, respectively, where *r, s* ∈ {1, 2}. The value in the lower-right entry is the total number of pixels in the images.

Except for the error matrix, the associated accuracy estimates are used, including the producer's accuracy, consumer's accuracy, overall accuracy and Kappa coefficient. Table 3 also gives the values of those measures. In conclusion, one would anticipate a high degree of accuracy in the segmented results from the proposed algorithm. From Table 3, Kappa coefficients are calculated as 0.92. According to the general interpretation rules for thematic accuracy assessment, the Kappa coefficients 0.81–1.00 can be interpreted as almost perfect [25].

Another scheme for the accurate assessment of the developed segmentation algorithm is based on the degree to which the outlines of segmented homogeneous regions match their alternatives delineating the real regions, which is measured by the count of pixels of extracted outlines lying on the buffer zone around the real outlines of homogenous regions [9].

**Figure 6.**
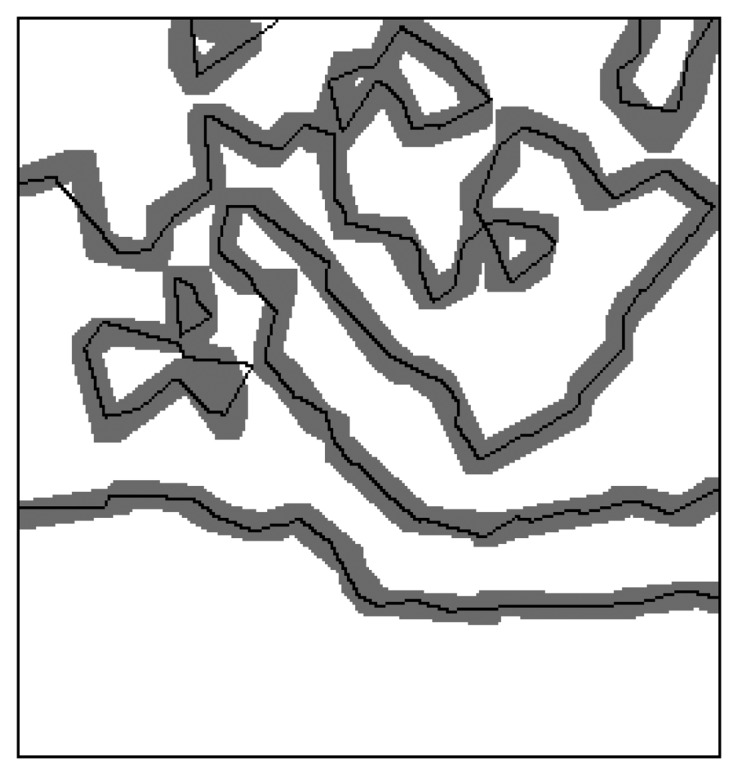
Extracted outlines overlaid on the buffer zones around the outlines of real regions.

Figure 6 shows the extracted outlines of the extracted oil spill regions laid on the buffer zones with 4 pixels width around their real outlines at each side, in which the gray zones are buffer zones and the black lines are the outlines of segmented homogenous regions. It can be observed that almost all extracted outlines lay on the buffer zones.

Table 4 gives the percents of the outlines for extracted oil spill regions on each buffer layer, where *B*_0_ means the percent of the outlines exactly matching the outlines for the real oil spill regions in the synthesizing images. *B_i_*'s, here *i* = 1, 2, 3, 4, represent the percents of the extracted outlines of oil spill regions lying on the *i*'th buffer layer of the real outlines. The Table also gives the accumulated percents *Σ_i_* = *B*_0_ + *B*_1_+ … + *B_i_*. It can be seen from the Table that over 90% of the outlines of segmented homogenous regions are within the buffer zone with two pixel width around the outlines of real homogenous regions and almost all outlines (around 99%) of segmented homogenous regions are in the buffer zone with four pixel width around the outlines of real homogenous regions.

The results from above two accuracy analyses schemes manifest that the proposed algorithm extracts the shape and distribution parameters features of oil spill regions efficiently and effectively.

### 3.2. Real SAR Imagery

**Figure 7.**
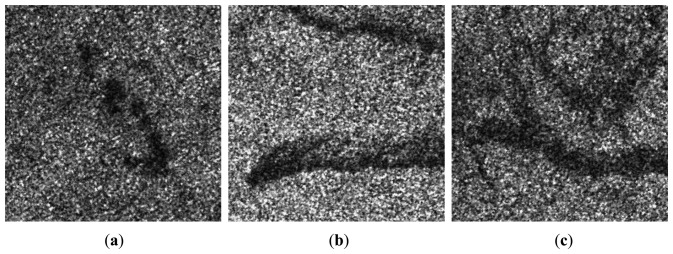
Real 4-looks SAR intensity images in which the dark areas are oil spills.

**Figure 8.**
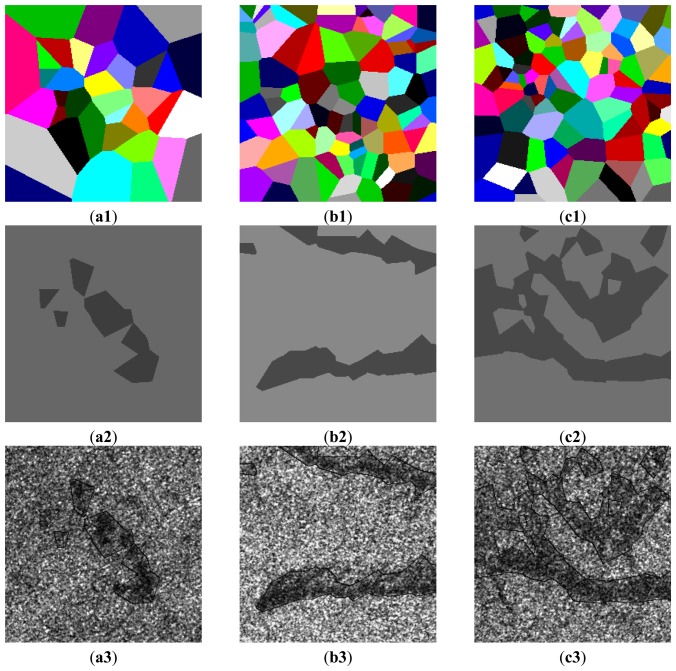
Final partition (**a1**), (**b1**), (**c1**) and segmentation (**a2**), (**b2**), (**c2**) of testing images. Darker regions in (**a2**), (**b2**), (**c2**) indicate oil spills. Overlaying the outlines of extracted darker regions on test images (**a3**), (**b3**), (**c3**).

Figure 7 shows real SAR intensity images of RADARSAT-I/II HH polarization with oil slicks which appears darker compared to the surrounding waters. The initial partitions of image domain ***D*** are carried out by the Voronoi tessellation, in which the number of generating points *m*_0_ is drawn from the Poisson distribution with the mean 96 and the locations of *m*_0_ generating points are drawn from ***D*** uniformly. The initial segmentation is performed by randomly assigning a label to each polygon in the initial partition of ***D*** from the Bernoulli distribution with probabilities *p_j_* = 1/2, where *j* = {1, …, *m*_0_}. It is found that there is no obvious impact of the initial segmentation on the finial segmentation result. Figure 8a1, b1, c1 show the results of the final partitions of ***D*** with 36, 95 and 96 polygons, respectively. Figure 8a2, b2, c2 show the results of the optimal segmentation in terms of the MAP estimation after 4,000 iterations, where the tone for each region is represented by its estimated mean.

Figure 8a3, b3, c3 show the overlay of the outlines in black on the real SAR intensity images. Visually, the segmented regions match their real regions well. Table 5 summarizes estimated *α*_1,2_ and *β*_1,2_ corresponding to the segmented dark spot and sea regions.

**Figure 9.**
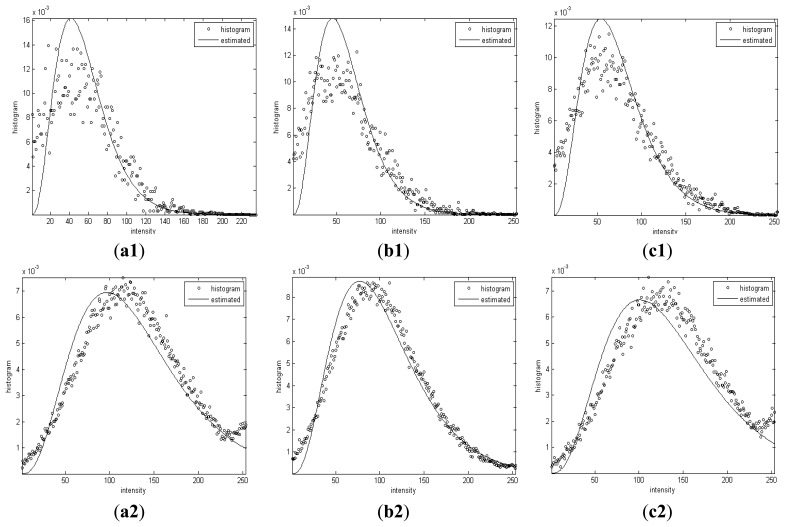
The histogram for two segmented homogeneous regions and gamma distribution curves with estimated parameters.

Figure 9 shows the histogram of intensities and Gamma distributions with the estimated shape and scale parameters of the segmented dark spots and sea background.

It can be observed that the histograms of intensities in each segmented homogenous region are coincident with the Gamma distribution curves with *α* and *β* derived by the proposed algorithm.

## 4. Conclusions

This paper presents a new segmentation-based approach to the feature extraction of oil spills from SAR intensity images, including their area and distribution parameters. The proposed segmentation algorithm is statistical region-based, which combines the Voronoi tessellation, Bayesian inference and reversible Jump Markov chain Monte Carlo (RJMCMC) methods. The Voronoi tessellation has been widely used to characterize models of many natural phenomena or processes in crystallography, metallography, physics, astrophysics, biology, ecology, geology, geography, *etc.*

In this paper the technique is introduced to design a region-based segmentation algorithm for oil spill feature extraction. By region, it means that Voronoi tessellation is used to partition the image domain into sub-regions (polygons) corresponding to components of oil spill regions or their surroundings (waters). Therefore, the segmentation of SAR intensity image for the purposes of oil spill feature extraction is completed by labeling those polygons as oil spills or water and thus modeled as a label field. By statistical, it means that each region (oil spill or water) is statistically homogenous, which is characterized by a Gamma distribution. Under the Bayesian inference paradigm, the label field for segmentation and distributing parameter can be expressed as a posterior conditional on a given SAR intensity image. The RJMCMC

method is employed to simulate the conditional posterior distribution. The results of testing the proposed approach on both real and synthesizing SAR intensity images demonstrate that it can extract the area and distribution parameters for oil spills with high accuracy and is promising.

## Figures and Tables

**Table 1.** Constants used in the experiment.

***λ***	***b***	***μ****_α_*	***σ****_α_*	***μ****_β_*	***σ****_β_*	***ε****_α_*	***ε****_β_*	***T***
96	1	4	1	32	8	0.5	1	4,000

**Table 2.** Estimated model parameters and their errors.

	***α***	***e****_α_* **(%)**	***β***	***e****_β_***(%)**
C1	4.04	1.0	27.37	2.25
C2	3.95	1.25	18.56	3.11

**Table 3.** Error matrix and statistical measurements.

	***C*_1_**	***C*_2_**	**Σ*C****_s_*	**Producer**'**s Accuracy (%)**
*C*_1_	39,908	1,448	41,356	94.13
*C*_2_.	971	23,209	24,180	97.61
Σ*C._r_*	40,879	24,657	65,536	Overall accuracy (%)
User's accuracy (%)	95.98	96.95	0.92	96.3

**Table 4.** Percentage of extracted outlines in buffer zones.

***B*_0_(%)**	***B*_1_/*Σ*_1_(%)**	***B*_2_/*Σ*_2_(%)**	**B_3_/*Σ*_3_(%)**	**B_4_/*Σ*_4_(%)**
35.7	39.6/75.3	15.6/90.9	5.7/96.6	1.7/98.3

**Table 5.** Estimated shape and scale parameters.

**Image**	***α*_1_**	***α*_2_**	***β*_1_**	***β*_2_**
a	5.00	3.22	28.27	23.11
b	4.06	2.44	28.64	34.02
c	4.72	2.67	31.02	32.44
